# Thickness-Dependent Differential Reflectance Spectra of Monolayer and Few-Layer MoS_2_, MoSe_2_, WS_2_ and WSe_2_

**DOI:** 10.3390/nano8090725

**Published:** 2018-09-14

**Authors:** Yue Niu, Sergio Gonzalez-Abad, Riccardo Frisenda, Philipp Marauhn, Matthias Drüppel, Patricia Gant, Robert Schmidt, Najme S. Taghavi, David Barcons, Aday J. Molina-Mendoza, Steffen Michaelis de Vasconcellos, Rudolf Bratschitsch, David Perez De Lara, Michael Rohlfing, Andres Castellanos-Gomez

**Affiliations:** 1 National Center for International Research on Green Optoelectronics & Guangdong Provincial Key Laboratory of Optical Information Materials and Technology, Institute of Electronic Paper Displays, South China Academy of Advanced Optoelectronics, South China Normal University, Guangzhou 510006, China; niuyue18401@163.com; 2 Instituto Madrileño de Estudios Avanzados en Nanociencia (IMDEA Nanociencia), Campus de Cantoblanco, E-28049 Madrid, Spain; sgonzaleza@indra.es (S.G.-A.); david.barcons@icfo.eu (D.B.); david.perezdelara@imdea.org (D.P.D.L.); 3 National Key Laboratory of Science and Technology on Advanced Composites in Special Environments, Harbin Institute of Technology, Harbin 150001, China; 4 Materials Science Factory, Instituto de Ciencia de Materiales de Madrid (ICMM), Consejo Superior de Investigaciones Científicas (CSIC), Sor Juana Inés de la Cruz 3, 28049 Madrid, Spain; riccardo.frisenda@imdea.org (R.F.); patricia.gant@csic.es (P.G.); najmehtaghavi80@gmail.com (N.S.T.); 5 Institute of Solid-state Theory, University of Münster, 48149 Münster, Germany; p_mara01@uni-muenster.de (P.M.); drueppel.matthias@gmail.com (M.D.); michael.rohlfing@uni-muenster.de (M.R.); 6 Institute of Physics and Center for Nanotechnology, University of Münster, 48149 Münster, Germany; Robert.Schmidt@uni-muenster.de (R.S.); michaelis@uni-muenster.de (S.M.d.V.); bratschi@uni-muenster.de (R.B.); 7 Faculty of Physics, Khaje Nasir Toosi University of Technology (KNTU), Tehrān 19697 64499, Iran; 8 Institute of Photonics, Vienna University of Technology, Gusshausstrasse 27–29, 1040 Vienna, Austria; aday.molina-mendoza@tuwien.ac.at

**Keywords:** 2D materials, transition metal dichalcogenides (TMDCs), MoS_2_, MoSe_2_, WS_2_, WSe_2_, optical properties, differential reflectance

## Abstract

The research field of two dimensional (2D) materials strongly relies on optical microscopy characterization tools to identify atomically thin materials and to determine their number of layers. Moreover, optical microscopy-based techniques opened the door to study the optical properties of these nanomaterials. We presented a comprehensive study of the differential reflectance spectra of 2D semiconducting transition metal dichalcogenides (TMDCs), MoS_2_, MoSe_2_, WS_2_, and WSe_2_, with thickness ranging from one layer up to six layers. We analyzed the thickness-dependent energy of the different excitonic features, indicating the change in the band structure of the different TMDC materials with the number of layers. Our work provided a route to employ differential reflectance spectroscopy for determining the number of layers of MoS_2_, MoSe_2_, WS_2_, and WSe_2_.

## 1. Introduction

The isolation of atomically thin semiconducting transition metal dichalcogenides (TMDCs) by mechanical exfoliation of bulk layered crystals, aroused the interest of the nanoscience and nanotechnology community on these 2D semiconductors [1,2,3,4,5,6,7]. These materials have a band gap within the visible part of the spectrum, bridging the gap between graphene (zero-gap semiconductor) and hexagonal boron nitride (wide-gap semiconductor). Recently, the band gap of semiconductor TMDCs has been exploited to fabricate optoelectronic devices, such as photodetectors [8,9,10,11,12,13,14] and solar cells [15,16,17,18,19]. Photoluminescence studies also demonstrated that a reduction in thickness has a strong effect on the band structure of MoS_2_ and other semiconductor TMDCs, including a change in the band gap and a thickness mediated direct-to-indirect band gap crossover [20,21,22,23,24]. The thickness dependent band gap can have a strong influence on other electrical [25] and optical properties, such as the absorption [25,26,27], and it has been also exploited to fabricate photodetectors where their spectral bandwidth is determined by the number of layers of the semiconductor channel [9,11]. However, the determination of the intrinsic quantum efficiency and the photoresponse of photodetectors based on semiconducting TMDCs, requires a comprehensive study of their reflectance and/or transmittance with different numbers of layers in a wide spectral range, which is still lacking.

We systematically study the differential reflectance of single- and few-layer MoS_2_, MoSe_2_, WS_2_, and WSe_2_, from the near-infrared (1.4 eV) to the near-ultraviolet (3.0 eV). The differential reflectance spectra show prominent features due to excitons, and the thickness dependence of these excitonic features is analyzed. 

## 2. Materials and Method

We prepared MoS_2_, MoSe_2_, WS_2_, and WSe_2_ nanosheets by mechanical exfoliation with blue Nitto tape (Nitto Denko Co., Tokyo, Japan, SPV 224P), on commercially available polydimethylsiloxane substrates (Gel-Film from Gelpak®, Hayward, CA, USA). MoSe_2_, WS_2_, and WSe_2_ bulk crystals were synthetic (grown by the vapor transport method), with approximate dimensions of 10 × 10 × 0.2 mm^3^, whilst the MoS_2_ material employed in this work was a naturally occurring molybdenite crystal (Moly Hill mine, QC, Canada), with approximate dimensions of 50 × 50 × 3 mm^3^. All the materials studied in the main text were 2H polytype. In the supporting information, we also include results obtained for mechanically exfoliated natural 3R-MoS_2_ (Mont St. Hilaire, QC, Canada), which occurs in the form of micro-crystals, with an area of 0.5 × 0.5 mm^2^ on the surface of a quartz mineral. Few-layer flakes were identified under an optical microscope (Nikon Eclipse CI, Tokyo, Japan), and the number of layers was determined by their opacity in transmission mode. The optical properties of the nanosheets were studied with a home-built micro-reflectance/transmittance setup, described in detail in Reference [28].

The calculations of the absorption spectra were conducted using the *GW*-BSE method, within the LDA + *GdW* approximation [29]. The dielectric screening was implemented by an atom-resolved model function, based on the random phase approximation. For the structural parameters, we used experimental values, as reported in Reference [30] (with *a* = 3.160 Å for MoS_2_ and *a* = 3.299 Å for MoSe_2_) and Reference [31] (with *a* = 3.155 Å for WS_2_ and *a* = 3.286 Å for WSe_2_). We started with a density functional theory (DFT) calculation within the local density approximation (LDA), using a basis set of localized Gaussian orbitals and norm-conserving pseudopotentials, which also included spin-orbit interaction. The resulting wave functions and energies were used for a subsequent *GdW* calculation, fully considering spin-orbit interaction. For the BSE calculations, we used a 24 × 24 × 1 *k*-point grid for the mono- and bi-layers, and an 18 × 18 × 3 *k*-point grid for the bulk crystals. Notably, we used identical meshes for both the quasiparticle corrections and the electron-hole interaction, so no interpolation scheme was needed. The number of valence and conduction bands in the BSE Hamiltonian were doubled, when going from the monolayer (four/six) to the bilayer and bulk crystals (eight/twelve). A detailed analysis of the convergence of the presented calculation is found in the Supporting Information. For all absorption spectra, an artificial broadening of 35 meV was applied. 

## 3. Results and Discussion

Single- and few-layer MoS_2_, MoSe_2_, WS_2_, and WSe_2_ samples were fabricated by mechanical exfoliation of bulk layered crystals onto a polydimethylsiloxane (PDMS) substrate (Gelfilm from Gelpak®, Hayward, CA, USA, as described in Reference [32]; see the Supporting Information for results obtained with other substrates). We refer the reader to the Materials and Methods section, for more details about the sample fabrication. Moreover, all results shown in the main text were obtained for the 2H polytype, which is the most common in this family of materials. We refer the reader to the Supporting Information, for a comparison between the 2H- and 3R-MoS_2_ polytypes. 

**Figure 1:**
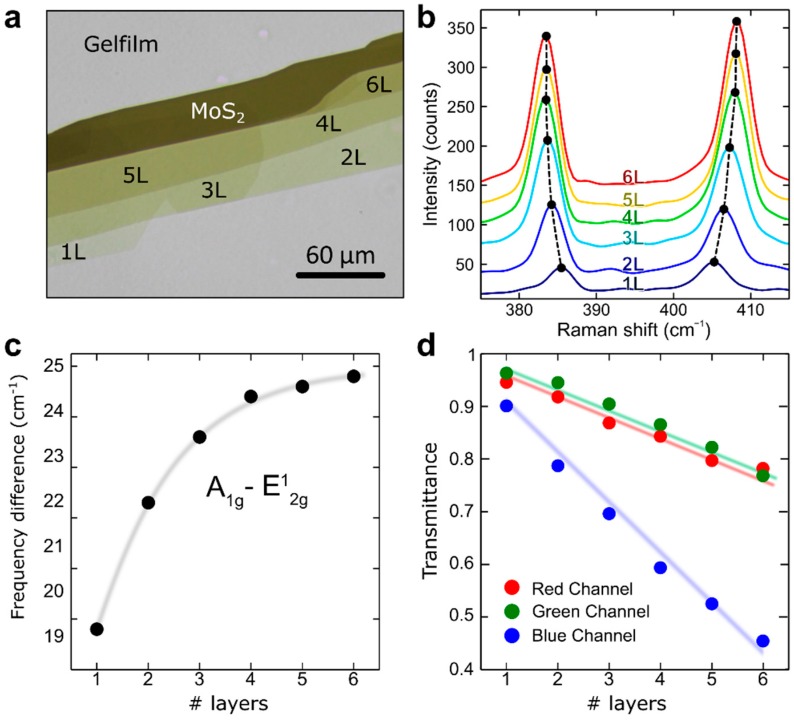
(**a**) Transmission mode optical image of a mechanically exfoliated MoS_2_ flake on polydimethylsiloxane (PDMS) substrate; (**b**) Raman spectra measured on the different regions of the flakes; The thickness of the flake can be determined from the Raman shift difference between the *A*_1g_ and *E*_2g_ lines, shown in panel (**c**). Note that a flake-to-flake variation of up to ~0.5 cm^−1^ can be found in the exfoliated flakes, and it would be the main cause of uncertainty in thickness determination through Raman spectroscopy; (**d**) Transmittance of the MoS_2_ flake (extracted from the red, green, and blue channels of the transmission mode optical images), as a function of the number of layers.

Figure 1a shows a transmission mode optical microscopy image of an exfoliated MoS_2_ flake, displaying regions with different numbers of layers, as determined from the position of the *E*_2g_ and *A*_1g_ lines in their Raman spectra (Figure 1b) [33,34]. Similar optical microscopy images of MoSe_2_, WS_2_, and WSe_2_ samples can be found in the Supporting Information. The quantitative analysis of the red, green, and blue channels of the transmission mode optical images, has recently been proven to be an effective alternative way to determine the number of layers of TMDCs (see description in Reference [35] and Supporting Information in Reference [36]). Figure 1c shows the transmittance *T/T*_0_ (*T*: intensity of the light transmitted through the flake, *T*_0_: intensity of the light transmitted through the substrate), extracted from the different regions of the transmission mode optical image shown in Figure 1a. The blue channel showed the largest thickness dependence. Thus, it can be very useful to determine the number of layers. The blue channel transmittance dropped monotonically by ~9% per MoS_2_ layer, in good agreement with the results reported in Reference [35]. We statistically analyzed the blue channel transmittance of 200 MoS_2_ flakes to get insight of the flake-to-flake variation, finding a typical value of 2–5%, allowing for the accurate determination of the flake thickness, despite the uncertainty introduced by these flake-to-flake variations. A more comprehensive statistical analysis of the blue channel transmittance in the whole family of TMDCs will be published elsewhere, as it lies out of the scope of the current manuscript.

We also found that the blue channel transmittance showed a strong thickness dependence for other TMDCs studied: WS_2_, WSe_2_, or MoSe_2_ (see the Supporting Information for an analogue of Figure 1c, for these materials). This strong thickness dependence of the blue channel transmittance, might be especially relevant to determine the number of layers of relatively thick MoS_2_ multilayers, as Raman spectroscopy is only accurate in determining layers thinner than 4 layers. The Raman shift difference between the *E*_2g_ and *A*_1g_, quickly saturates for flakes thicker than 4 layers, see Reference [33]. Furthermore, for WS_2_, WSe_2_ or MoSe_2_, it is not trivial to determine the number of layers with Raman spectroscopy, as one might need a high resolution Raman system or a system capable to resolve shear Raman modes occurring at low Raman shifts [37,38,39,40,41,42] (see the Supporting Information to see the thickness dependent Raman spectra of WS_2_, WSe_2_ or MoSe_2_). 

The optical spectra of the fabricated flakes were characterized using a homebuilt micro-reflectance and transmittance setup. We refer the reader to References [28,43], for details on this experimental setup. Briefly, the setup consisted of a Motic BA310 metallurgical microscope equipped with a 50× objective (0.55 numerical aperture and 8.2 mm of working distance), supplemented with a modified trinocular port, which sends part of the reflected light to a multimode fiber-coupled charge coupled device (CCD) spectrometer (Thorlabs CCS200/M, Newton, NJ, USA) to be analyzed. The system can be used to measure differential reflectance and transmittance, with a lateral resolution of ~1 µm. In the Supplementary Information, the reader will find a schematic diagram of the experimental setup configurations used for differential reflectance and transmittance experiments. In the main text, we showed the results of differential reflectance measurements, and we refer the reader to the Supporting Information, for a comparison between differential reflectance and transmittance measurements acquired on the same sample.

**Figure 2:**
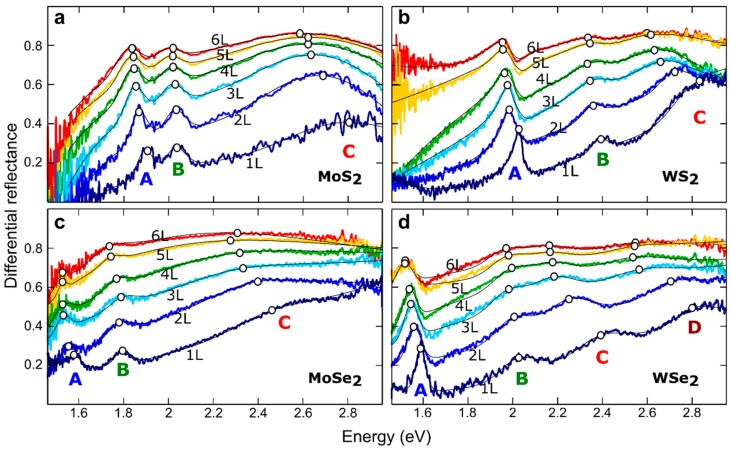
Differential reflectance spectra, measured as a function of the number of layers for (**a**) MoS_2_, (**b**) WS_2_, (**c**) MoSe_2_, and (**d**) WSe_2_. The spectra have been fitted to a sum of Lorentzian/Gaussian peaks (solid thin black lines), to determine the position of the different excitonic features (highlighted by white circles).

The differential reflectance spectrum was calculated as (*R* − *R*_0_)/*R*, and it is related to the absorption coefficient of the material α(λ) as shown in References [26,44]

(1) $$\frac{R-R_{0}}{R}=\frac{4n}{{n_{0}}^{2}-1}\alpha \left(\lambda \right)$$

where *R* is the intensity reflected by the flake, *R*_0_ the intensity reflected by the substrate, *n* is the refractive index of the flake under study, and *n*_0_ is the refractive index of the substrate. Figure 2 shows the differential reflectance spectra measured on the single- and few-layer regions, for the different semiconductor TMDCs studied. The spectra acquired for this family of 2D materials showed overall similar features: Pronounced peaks corresponding to the generation of excitons. The exact energy at which these peaks appeared is material-to-material dependent, because those features were determined by the band structures of these different compounds. The exciton peaks in Figure 2 are labelled A, B, and C (and D for WSe_2_), following the nomenclature employed in the literature to name the different excitons in semiconducting TMDCs [20,21,22]. The A exciton, occurring near the absorption band edge, corresponded to direct band gap transitions at the *K* point in the Brillouin zone [20,21,22]. This feature is the most studied one, as it is also the dominant one in photoluminescence spectra. Close to the A exciton peak, at slightly higher energy, the transition metal dichalcogenides showed another prominent peak in their differential reflectance spectra, corresponding to another direct band gap transition at the *K* point, but at higher energy, which yielded the creation of the so-called B excitons. For monolayer TMDCs, the origin of this higher energy transition at the *K* point was related to the splitting of the valence band, due to the spin-orbit interaction. For multilayer systems, the splitting of the valence band was driven by a combination of spin-orbit and interlayer interaction. 

Apart from the narrow A and B exciton peaks, the differential reflectance spectra of MoS_2_, MoSe_2_, and WS_2_, also showed other broader spectroscopic features in an energy range from 2.5 eV to 2.9 eV (referred to as the C exciton peak). This was due to singularities in the joint density of states between the first valence and conduction bands, in a circle around the *Γ* point (into the local minimum of the lowest conduction band between *Γ* and *K*), which led to multiple optical transitions to nearly degenerate in energy [26,27,45,46,47,48,49]. Regarding WSe_2_, we found that instead of just one broad C exciton feature, two features labeled C and D, were reported in recent absorption measurements [49]. Whilst the C exciton of WSe_2_ consisted of several transitions along the *Γ*-*K* direction, between the highest valence and lowest conduction bands, the D exciton had the largest contributions from the spin-split lower valence band, into the lowest conduction band [49].

The differential reflectance spectra were fit to a sum of Gaussian/Lorentzian peaks, with a broad background to determine the peak position, width, and magnitude of the excitonic features, as a function of the number of layers for the different 2D semiconductor materials. The thin black lines in Figure 2 correspond to the resulting fits for the different measured spectra, and the empty circles highlight the energy values determined for the different excitons from the fits. We found that the flake-to-flake variation in the peak position was the most relevant source for uncertainty, in the analysis of the thickness dependent spectra. In the Supplementary Information, we compare the spectra acquired on 6 single-layers and 4 bilayers of MoS_2_, to illustrate the typical flake-to-flake variation in the spectra. The exciton positions can shift up to 10–15 meV, and the flake-to-flake variations in intensity can reach 5–10%.

**Figure 3:**
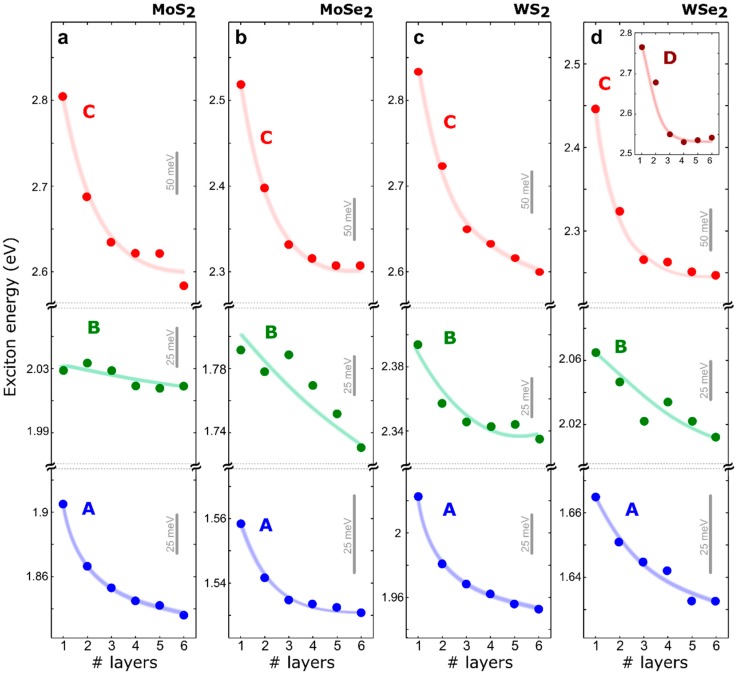
Thickness dependence of the exciton energies, extracted from the measured differential reflectance spectra of (**a**) MoS_2_, (**b**) WS_2_, (**c**) MoSe_2_, and (**d**) WSe_2_. The solid lines are guides to the eye.

To visualize the thickness dependence of the exciton energies, Figure 3 summarizes the determined exciton energies, as a function of the number of layers for MoS_2_, MoSe_2_, WS_2_, and WSe_2_. The A exciton peak redshifts as the thickness increases for all the studied materials, in agreement with previous photoluminescence results. The B exciton displayed a non-monotonic thickness dependence, which might arise from the moderate thickness dependence and the 10–15 meV flake-to-flake variation of the peak position. Overall, the B exciton feature also shifted to lower energy with the increasing number of layers. As discussed above, for single layers, the separation between the A and B exciton peaks is due to the spin-orbit splitting of the valence band. Therefore, the larger spin-orbit splitting induced by the heavier W atoms with respect to Mo atoms, is translated to a larger separation of the A and B features in the differential reflectance spectra of W-based TMDCs. Moreover, Se-based dichalcogenides exhibited a larger splitting between the A and B exciton peaks, than that of S-based dichalcogenides. Table 1 summarizes the values of the splitting between the A and B excitons for the single-layer TMDCs studied in this work and compares these values with theoretical values obtained through *ab initio* calculations (see the Supporting Information for more details about the calculations). The good agreement between predicted A-B splitting and the experimental values, confirms our theoretical calculations captured the essential features to describe the excitonic properties of TMDCs. Note that experimental values for the A-B splitting reported in the literature show variability [49,50,51], e.g., due to different experimental techniques and flake-to-flake variations due to small differences in strain, doping, and/or presence of defects. This explains the variation in discrepancies between our predicted and experimental AB-splittings.

Interestingly, we also found that the C exciton showed a prominent shift with the thickness, even more pronounced than that of the A exciton. Note that the number of works studying the C excitonic feature are still very scarce, as most experiments employ photoluminescence with green laser excitation (*E*~2 eV–2.3 eV) to observe the generated excitons. 

## 4. Conclusions

In summary, we presented a systematic study of the differential reflectance spectra MoS_2_, MoSe_2_, WS_2_, and WSe_2_, from the near-infrared (1.4 eV) to the near-ultraviolet (3.0 eV). The differential reflectance spectra showed prominent features due to the generation of excitons, and the energy at which these features appear depends on the thickness of the flakes, because of quantum confinement effects. We proposed employing a combination of a quantitative analysis of transmission mode optical images and differential reflectance measurements, as an alternative method to determine the number of layers. 

## Figures and Tables

**Table 1:** Comparison of the spin-orbit splitting extracted from the differential reflectance spectra, and those obtained from ab initio calculations, including spin-orbit interaction.

Material	Experimental A-B Splitting (meV)	Theoretical A-B Splitting (meV)
1L—MoS_2_	124 ± 5	152
1L—MoSe_2_	219 ± 10	218
1L—WS_2_	371 ± 5	420
1L—WSe_2_	398 ± 10	464

## Supplementary Materials

The following are available online at http://www.mdpi.com/2079-4991/8/9/725/s1, Figure S1: Transmission mode optical images of mechanically exfoliated transition metal dichalcogenides (TMDCs) onto polydimethylsiloxane (PDMS) substrates; Figure S2: Transmittance (extracted from the red, green, and blue channels of the transmission mode optical images), as a function of the number of layers; Figure S3: Blue channel transmittance measured on more than 200 MoS_2_ flakes with different numbers of layers (ranging from 1 layer to 4 layers); Figure S4: Comparison between the differential reflectance spectra measured for single-layer MoS_2_ on different substrates: glass, polycarbonate (PC), polypropylene (PP), and polydimethylsiloxane (PDMS); Figure S5: Reflection mode optical images of MoS_2_, WS_2_, MoSe_2_, and WSe_2_ after transfer onto SiO_2_/Si substrates (285 nm thick SiO_2_); Figure S6: Thickness dependence of the optical contrast measured for MoS_2_, WS_2_, MoSe_2_, and WSe_2_ deposited onto SiO_2_/Si substrates (285 nm thick SiO_2_); Figure S7: Raman spectra measured on MoS_2_, WS_2_, MoSe_2_, and WSe_2_ deposited onto PDMS substrates; Figure S8: Quantitative analysis of the Raman spectra measured on MoS_2_, WS_2_, MoSe_2_, and WSe_2_ deposited onto PDMS substrates; Figure S9: Comparison between differential reflectance and transmittance measurements, carried out on the same MoS_2_ flakes on PDMS; Figure S10: Differential reflectance intensity, measured from the differential reflectance spectra shown in Figure S6, at energies outside the excitonic resonance windows; Figure S11: Comparison between different methods to measure the optical properties of 2D materials (using 1L, 2L, and 3L MoS_2_ as testbeds); Figure S12: Comparison between the crystal structure of the 2H- and 3R-polytypes; Figure S13: Transmission mode optical images of 2H-MoS_2_ and 3R-MoS_2_ on PDMS; Figure S14: Transmittance (extracted from the red, green, and blue channels of the transmission mode optical images), as a function of the number of layers for 2H-MoS_2_ and 3R-MoS_2_; Figure S15: Differential reflectance spectra, measured as a function of the number of layers for 2H- and 3R-MoS_2_; Figure S16: Thickness dependence of the exciton energies, extracted from the differential reflectance spectra of 2H-MoS_2_ and 3R-MoS_2_; Figure S17: Convergence of the quasiparticle gap at the *K* point, with respect to the energy cutoff used in the LDA + *GdW* approach, for the representation of ε and *W*; Figure S18: Convergence of the A and B exciton for all four TMDCs, with respect to the *k*-point grid used in the BSE; Figure S19: Convergence of the A and B exciton for the bulk crystal of MoS_2_, with respect to the *k*-point grid applied in the BSE; Figure S20: Absorption spectra of MoS_2_, WS_2_, MoSe_2_, and WSe_2_, for all studied number of layers; Figure S21: Direct comparison between the exciton energies obtained experimentally, and those calculated for MoS_2_, WS_2_, MoSe_2_, and WSe_2_; Figure S22: Comparison between differential reflectance spectra acquired on different single-layer and bilayer MoS_2_ flakes, which illustrates how the flake-to-flake variation is the main source of uncertainty in the analysis of the thickness dependent spectra of TMDCs; Figure S23: Schematic of the experimental setup employed for the micro-reflectance and transmittance measurements on TMDCs.
