# Supplementary material for: Thickness-Dependent Differential Reflectance Spectra of Monolayer and Few-Layer MoS2, MoSe2, WS2 and WSe2
Source: Nanomaterials (Basel). 2018 Sep 14;8(9):725. doi: 10.3390/nano8090725 (PMC6163246; doi:10.3390/nano8090725)
Supplement: Supplementary file 1 [file nanomaterials-08-00725-s001.pdf]

Supporting information:

Thickness-Dependent Differential Reflectance Spectra of Monolayer and Few-Layer MoS_2, $MoSe_2$, WS_2 and WSe_2

Yue Niu [1,2,3], Sergio Gonzalez-Abad [2], Riccardo Frisenda [4], Philipp Marauhn [5], Matthias Drüppel [5], Patricia Gant [4], Robert Schmidt [6], Najme S. Taghavi [4,7], David Barcons [2], Aday J. Molina-Mendoza [8], Steffen Michaelis de Vasconcellos [6], Rudolf Bratschitsch [6], David Perez De Lara [2], Michael Rohlfing [5] and Andres Castellanos-Gomez [4,*]

[1] National Center for International Research on Green Optoelectronics & Guangdong Provincial Key Laboratory of Optical Information Materials and Technology, Institute of Electronic Paper Displays, South China Academy of Advanced Optoelectronics, South China Normal University, Guangzhou 510006, P. R. China.; niuyue18401@163.com

[2] Instituto Madrileño de Estudios Avanzados en Nanociencia (IMDEA Nanociencia), Campus de Cantoblanco, E-28049 Madrid, Spain; sgonzaleza@indra.es (S.G.-A.); david.barcons@icfo.eu (D.B.); david.perezdelara@imdea.org (D.P.D.L.)

[3] National Key Laboratory of Science and Technology on Advanced Composites in Special Environments, Harbin Institute of Technology, Harbin 150001, China

[4] Materials Science Factory, Instituto de Ciencia de Materiales de Madrid (ICMM), Consejo Superior de Investigaciones Científicas (CSIC), Sor Juana Inés de la Cruz 3, 28049 Madrid, Spain; riccardo.frisenda@imdea.org (R.F.); patricia.gant@csic.es (P.G.); najmehtaghavi80@gmail.com (N.S.T.)

[5] Institute of Solid-state Theory, University of Münster, 48149 Münster, Germany; p_mara01@uni-muenster.de (P.M.); drueppel.matthias@gmail.com (M.D.); michael.rohlfing@uni-muenster.de (M.R.)

[6] Institute of Physics and Center for Nanotechnology, University of Münster, 48149 Münster, Germany; Robert.Schmidt@uni-muenster.de (R.S.); michaelis@uni-muenster.de (S.M.d.V.); bratschi@uni-muenster.de (R.B.)

[7] Faculty of Physics, Khaje Nasir Toosi University of Technology (KNTU), Tehrān 19697 64499, Iran

[8] Institute of Photonics, Vienna University of Technology, Gusshausstrasse 27–29, 1040 Vienna, Austria; aday.molina-mendoza@tuwien.ac.at

* Correspondence: andres.castellanos@csic.es

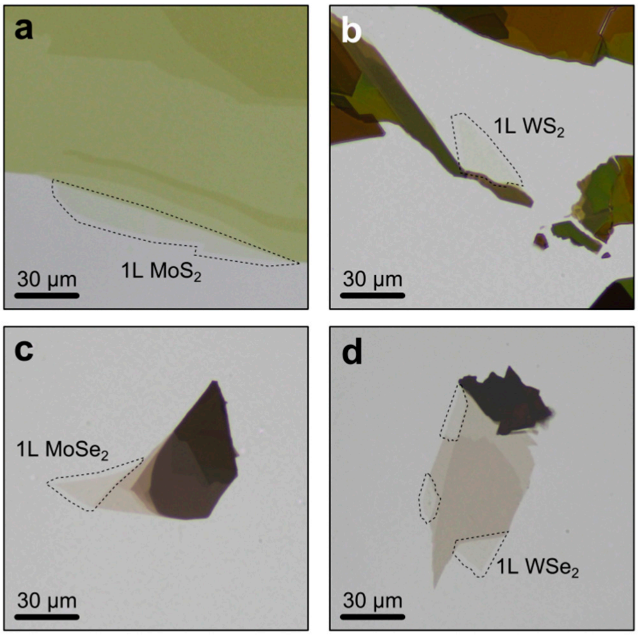

Figure S1. Transmission mode optical images of mechanically exfoliated TMDCs onto PDMS substrates. (a) MoS_2. (b) WS_2. (c) $MoSe_2$. (d) WSe_2. Single-layer areas have been highlighted with a dashed black line.

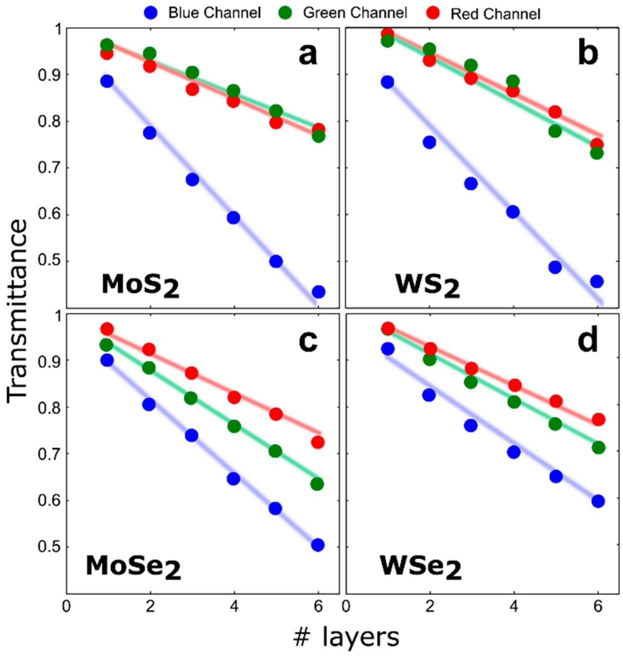

Figure S2. Trasmittance (extracted from the red, green and blue channels of the trasnmission mode optical images) as a function of the number of layers. (a) MoS_2. (b) WS_2. (c) $MoSe_2$. (d) WSe_2. The solid lines are guides to the eye.

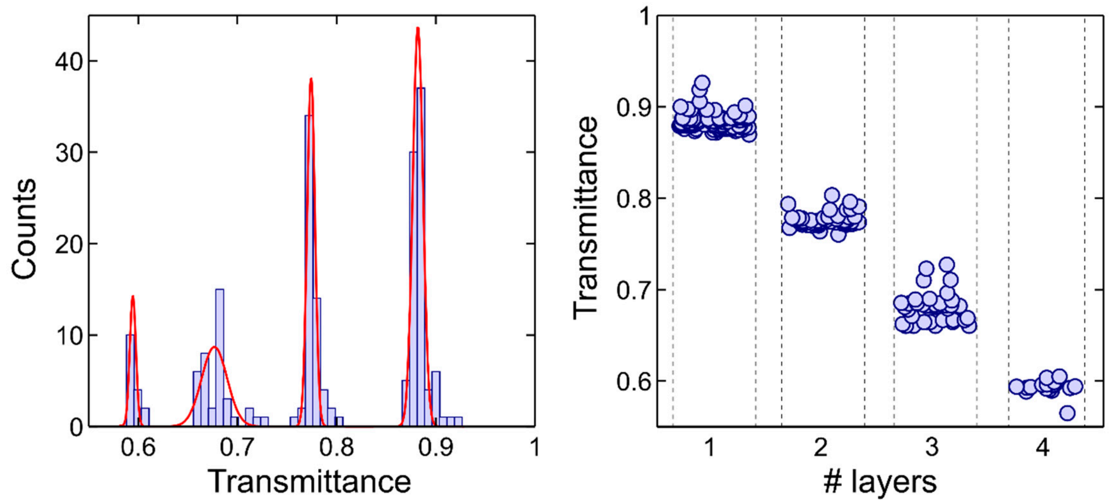

Figure S3. (a) Histogram of the blue channel transmittance measured on more than 200 MoS_2 flakes with different number of layers (ranging from 1 layer to 4 layers). The histogram has been fitted to a sum of 4 Gaussian curves. (b) Number of layers assigned from the transmittance of the blue channel of the same Mos_2 flakes shown in (a).

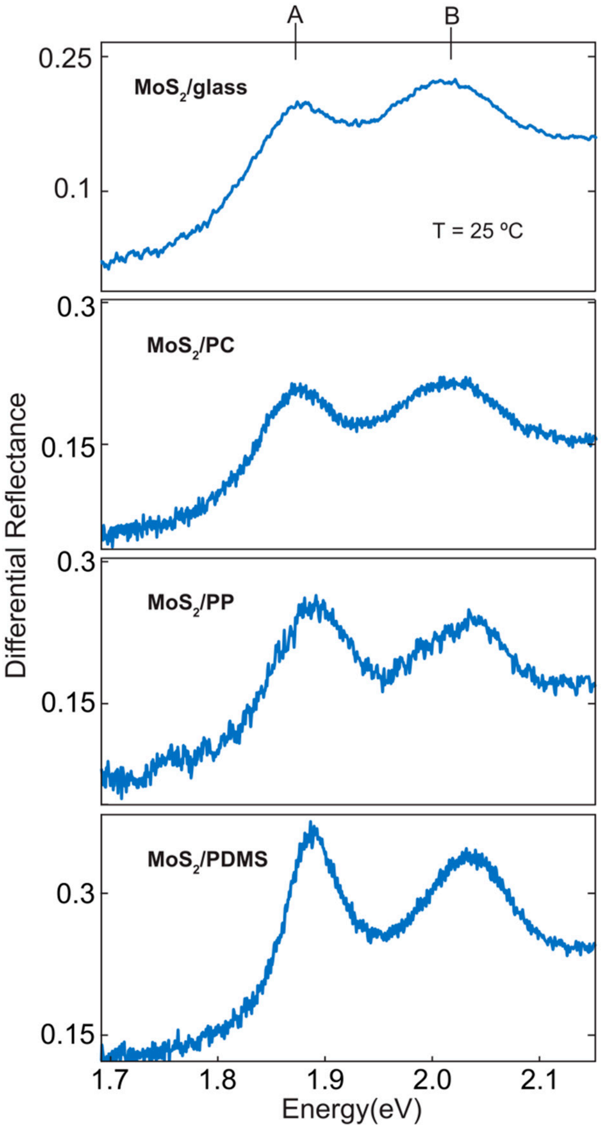

Figure S4. Comparison between the differential reflectance spectra measured for single-layer MoS_2 on different substrates: glass, polycarbonate (PC), polypropylene (PP)and poly-dimethil siloxane (PDMS).

One advantage of choosing PDMS as substrate for the characterization of TMDCs is that once the flakes are fully characterized they can be easily transferred to another substrate by means of an all-dry transfer method that exploits the viscoelasticity of PDMS to accomplish the transfer of the flake.[45] Figure S5 shows some examples of TMDC flakes that have been transferred from the PDMS substrate to a silicon substrate with a 285 nm SiO_2 capping layer, which is one of the standard substrates employed in many laboratories working with graphene and other 2D materials.

For 2D materials supported on SiO_2/Si substrates the quantitative analysis of their optical contrast (defined as $C = (I_{flake}-I_{subs})/(I_{flake}+I_{subs})$) is a common method to identify atomically thin flakes and to estimate their number of layers.[46–52] These analyses are typically carried out by acquiring reflection mode optical images while the illumination wavelength is selected by means of narrow bandpass filters[46,49,50], by hyperspectral imaging [39,53], or by using the micro-reflectance setup employed in this work.[32,33] Figure S6 shows a summary of the optical contrast spectra acquired for MoS_2, WS_2, $MoSe_2$ and WSe_2 flakes with different number of layers. Although this figure could be used as a guide to determine the number of layers of TMDCs exfoliated onto SiO_2/Si substrates, the difference in optical contrast spectra between layers with different thicknesses is more subtle than that measured onto the PDMS substrate by differential reflectance. Also the spectra show a skewed 'S' shape because of the interference color effect, due to the thin SiO_2 dielectric layer on top of the reflective silicon surface, which hampers the identification of the excitonic features that are superimposed (still visible on the MoS_2 flakes, Figure S6a). Therefore, these results illustrate that it is preferable to characterize the TMDCs on the PDMS substrate (by means of the combination of the quantitative analysis of the transmission mode optical images and the differential reflectance/transmittance) prior to their transfer to SiO_2/Si substrates.

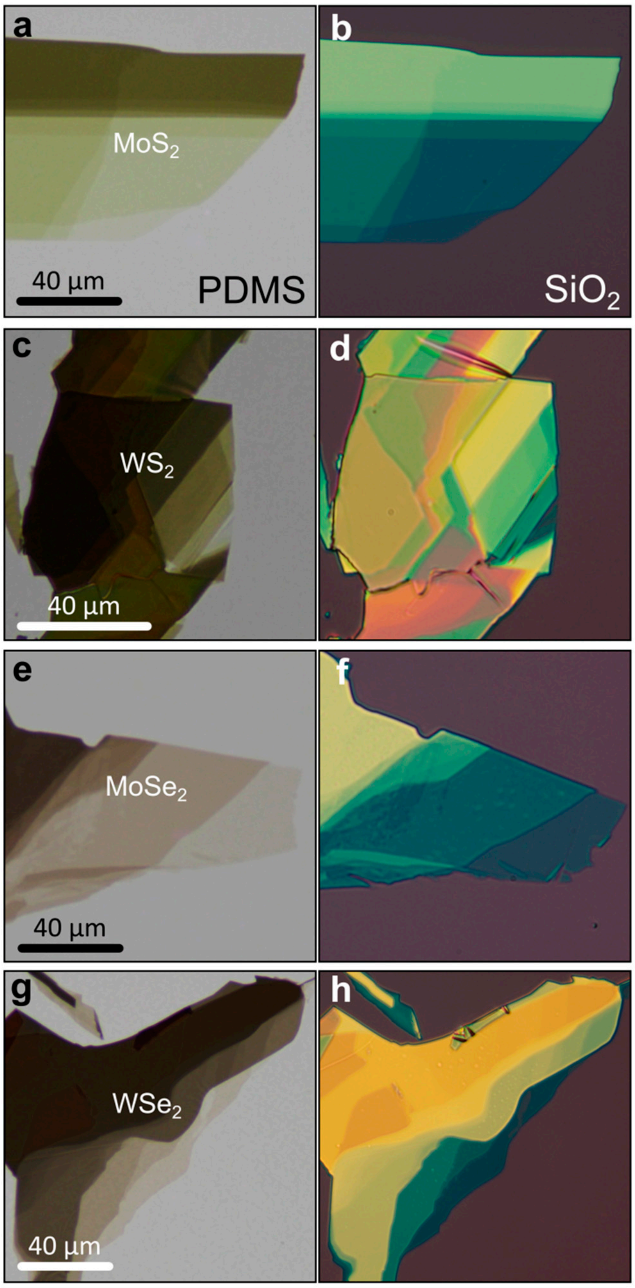

Figure S5. Transmission mode optical images (left panels) of MoS_2 (a), WS_2 (c), $MoSe_2$ (e) and WSe_2 (g) on PDMS substrates. Reflection mode optical images of the same flakes after transfer onto SiO_2/Si substrates (285 nm thick SiO_2): MoS_2 (b), WS_2 (d), $MoSe_2$ (f) and WSe_2 (h). Note: the images on SiO_2/Si substrates have been flipped horizontally to facilitate the comparison with the transmission mode images.

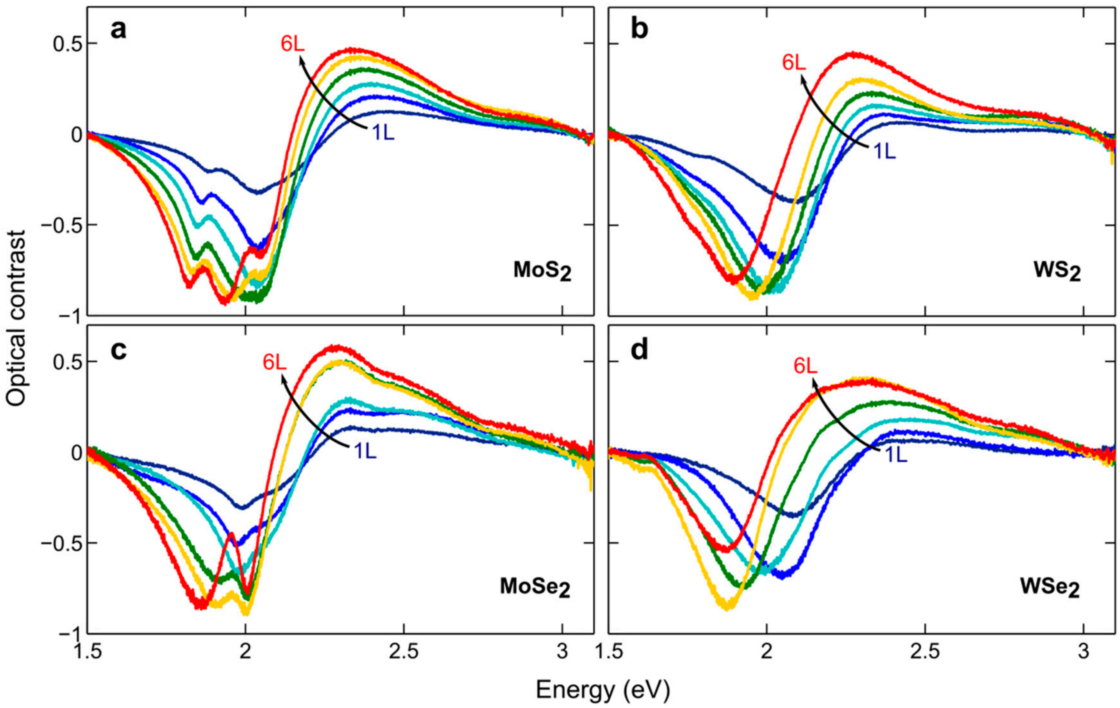

Figure S6. Thickness dependence of the optical contrast measured for MoS_2 (a), WS_2 (b), $MoSe_2$ (c) and WSe_2 (d) deposited onto SiO_2/Si substrates (285 nm thick SiO_2).

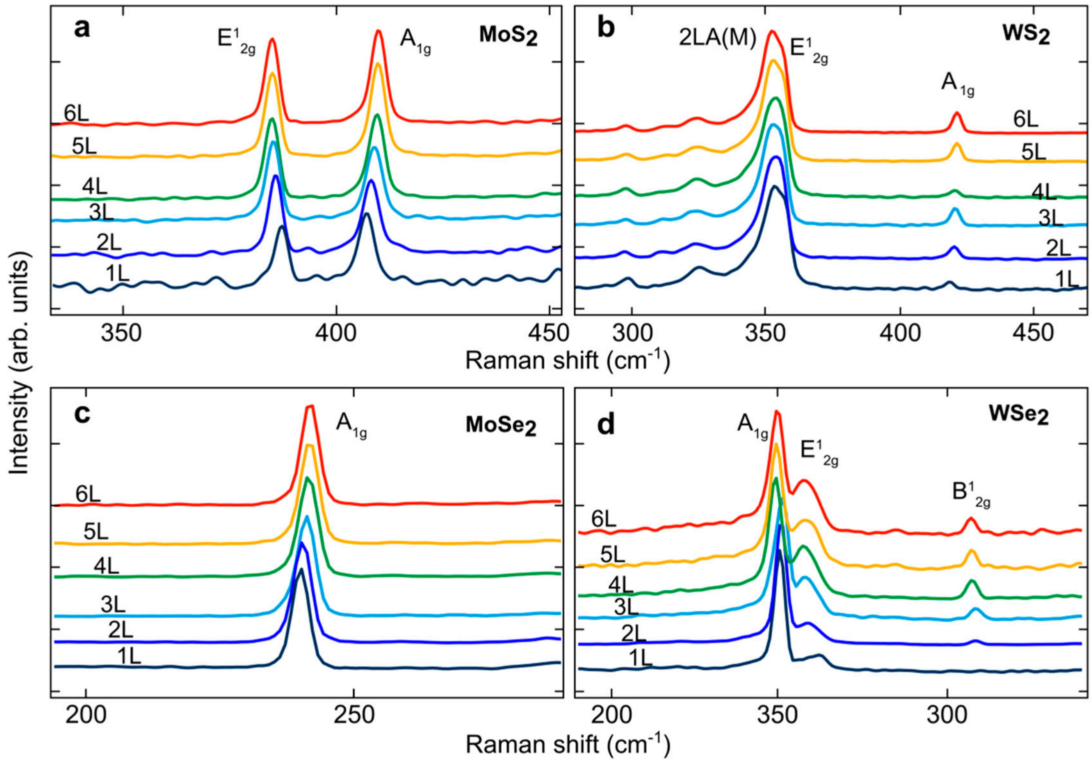

Figure S7. Raman spectra mesured on MoS_2 (a), WS_2 (b), $MoSe_2$ (c) and WSe_2 (d) deposited onto PDMS substrates.

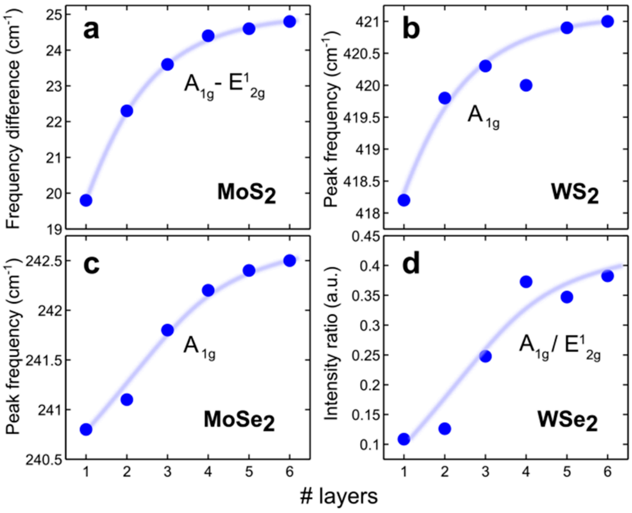

Figure S8. Quantitative analysis of the Raman spectra measured on MoS_2 (a), WS_2 (b), $MoSe_2$ (c) and WSe_2 (d) deposited onto PDMS substrates. The solid lines are guides to the eye.

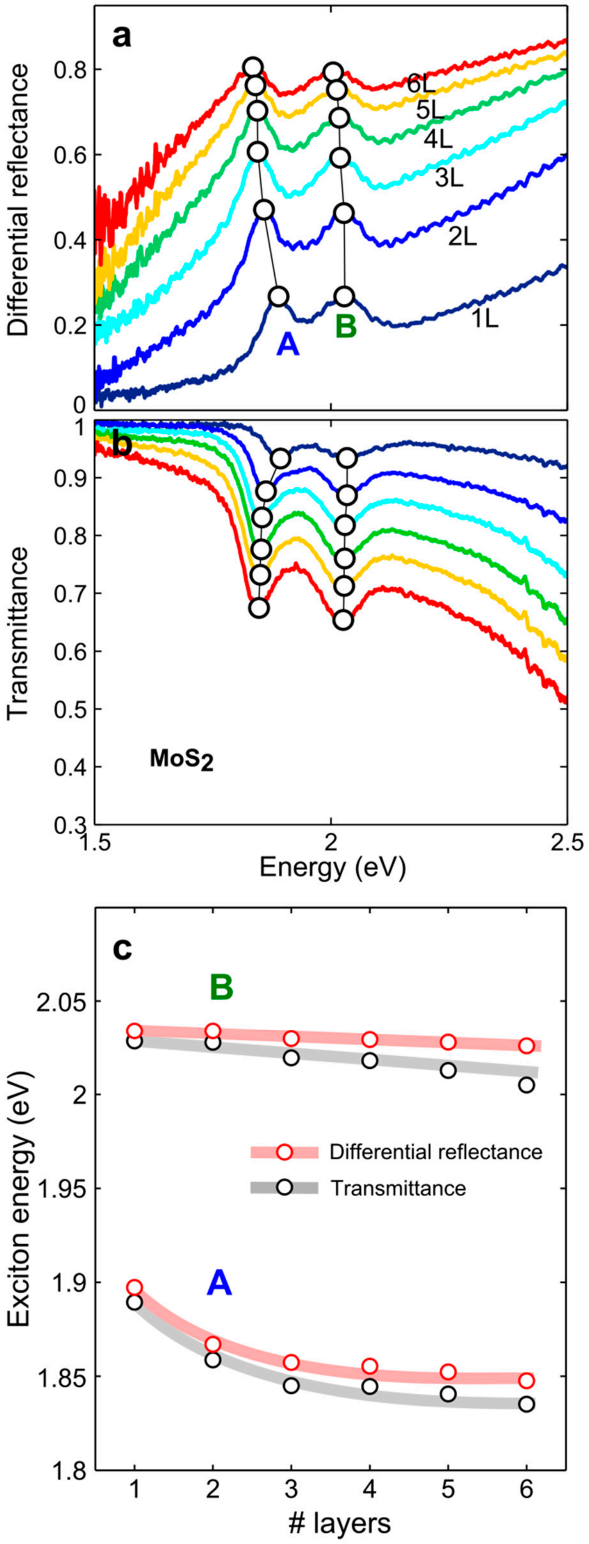

Figure S9. Comparison between differential reflectance (a) and transmittance (b) measurements carried out on the same MoS_2 flakes on PDMS. (c) Comparison between the exciton energies determined from differential reflectance and transmittance measurements. The slight variation between the two methods could be attributed to a slight increase of temperature of the substrate during the transmittance measurements (leading to a slight biaxial straining of the flakes).

Now we turn our attention to the differential reflectance spectra outside the energy window where the exciton resonances occur. We have found that the differential reflectance magnitude increases monotonically with the number of layers. The results are summarized in Figure 7, demonstrating that a quantitative analysis of epi-illumination images can be used to determine the thickness of the MoS_2, $MoSe_2$, WS_2 and WSe_2 samples. The quantitative analysis could be carried out by selecting the illumination wavelength with narrow-bandpass filters, typically used in most laboratories to enhance the optical contrast of 2D materials.

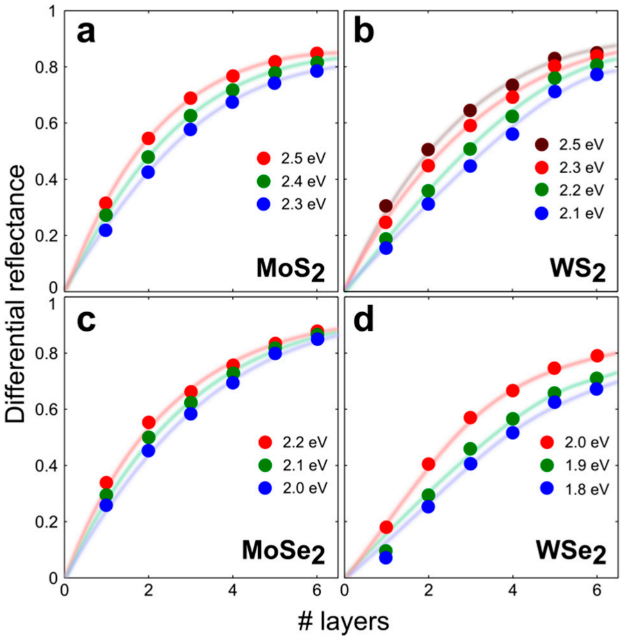

Figure S10. Differential reflectance intensity measured from the differential reflectance spectra shown in Figure S6 at energies outside the excitonic resonance windows: (a) MoS_2, (b) WS_2, (c) $MoSe_2$ and (d) WSe_2. The solid lines are guides to the eye.

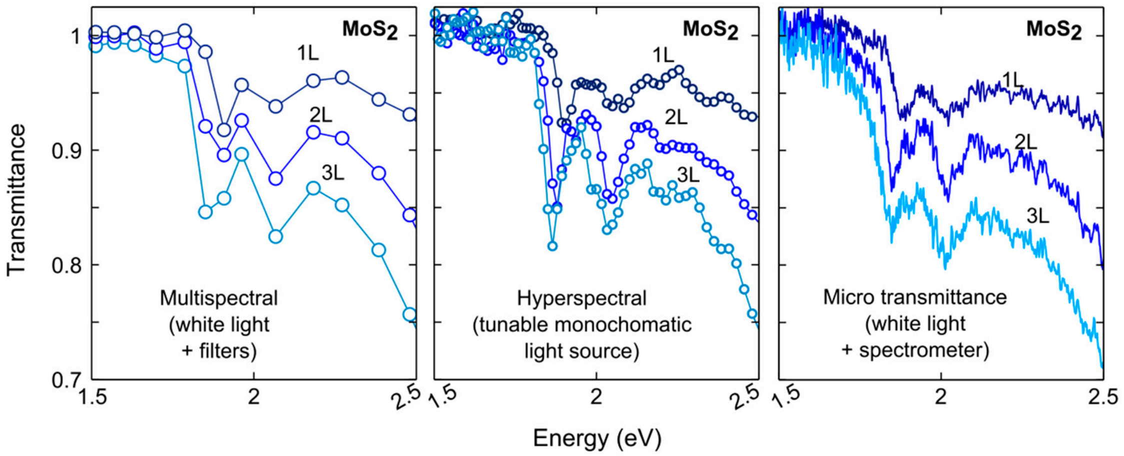

Figure S11. Comparison between different methods to measure the optical properties of 2D materials (using 1L, 2L and 3L MoS_2 as testbed). In the multispectral measurements narrow bandwidth filters are used to select the illumination wavelength. In the hyperspectral method the illumination is carried out through a white-light source connected to a monochromator. In the micro-transmittance measurement, we employ white light, which is collected through an optical fiber (acting as a confocal pinhole) and sent to a CCD spectrometer.

Figure S12 shows an artistic representation of the crystal structure of 2H- (Figure S11 up) and 3R- (Figure S12 down) MX_2 crystals. The 2H and 3R phase differ in the bulk crystals, since it arises from a different stacking of 2D layers, interacting by van der Waals forces. For instance, in MoS_2, the 2H phase presents unit cell parameters $a = b = 3.1625$ Å and c = 12.300 Å (space group $P6_3/mmc$), while the 3R phase presents unit cell parameters $a = b = 3.1607$ Å and c = 18.344 Å (space group *R3m*).[1]

The different stacking of the 2H and the 3R phase leads to slightly distinct band structures and, therefore, different excitonic phenomena. In the case of MoS_2, for example, the energy splitting of the top of the valence band at the $\overline{K}$ point is smaller for the 3R-MoS_2 (0.14 eV) than for the 2H-MoS_2 (0.17 eV), which is translated in different exciton splitting [2]. This different splitting can be observed in the differential reflectance spectra (Figure S14 and Figure S15). In Figure S13 we show optical microscopy images of mechanically exfoliated flakes of 2H- and 3R- MoS_2 mono- and few-layer crystals on a PDMS substrate, where no difference can be depicted between the two phases. Also, the transmittance extracted from transmission mode images seems very similar for both 2H and 3R polytypes (Figure S14). Therefore, the quantitative analysis of the A and B exciton energy difference seems the more reliable way to distinguish between the 2H and the 3R polytypes.

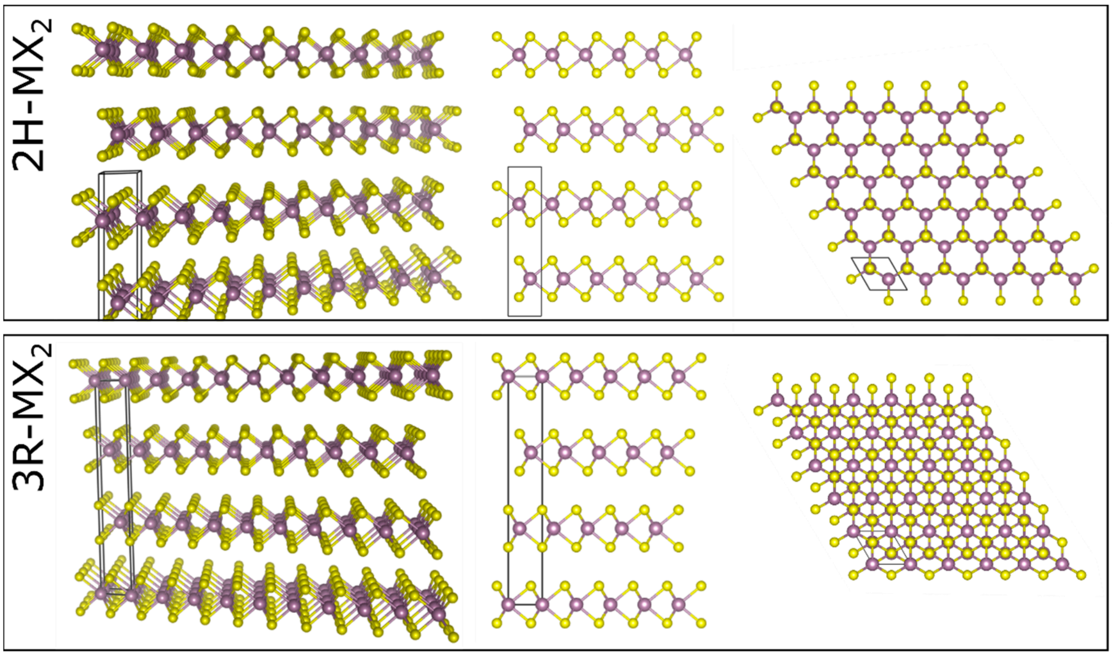

Figure S12. Comparison between the crystal structure of the 2H- and 3R- polytypes.

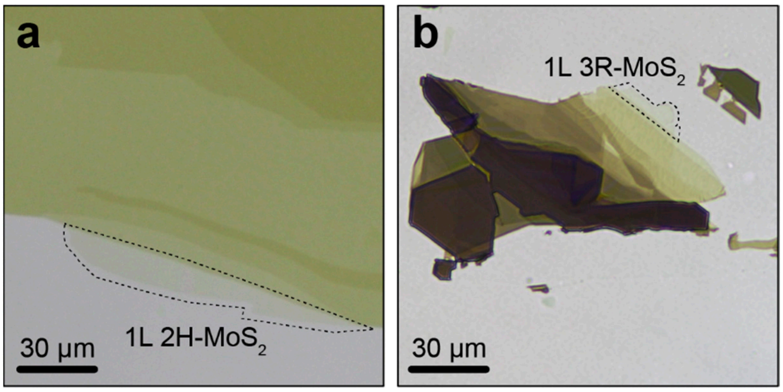

Figure S13. Transmission mode optical images of 2H-MoS_2 (a) and 3R-MoS_2 on PDMS. The dashed regions highlight the single-layer regions. Note that MoS_2 single-layers of 2H and 3R have the same structure and they only differ for multilayered stacks.

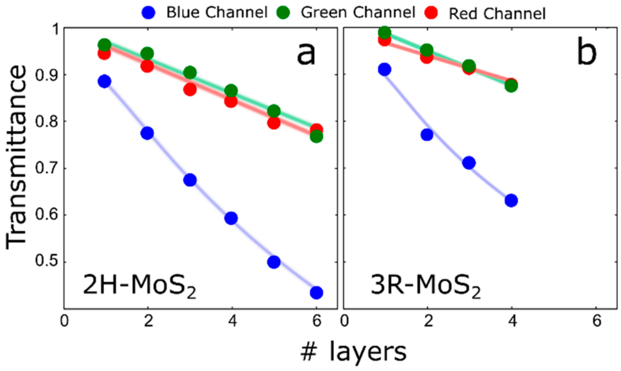

Figure S14. Transmittance (extracted from the red, green and blue channels of the trasnmission mode optical images) as a function of the number of layers for (a) 2H-MoS_2 (data

reproduced from Figure 1d to facilitate the comparison with the 3R polytype) and (b) 3R-MoS_2. The solid lines are guides to the eye.

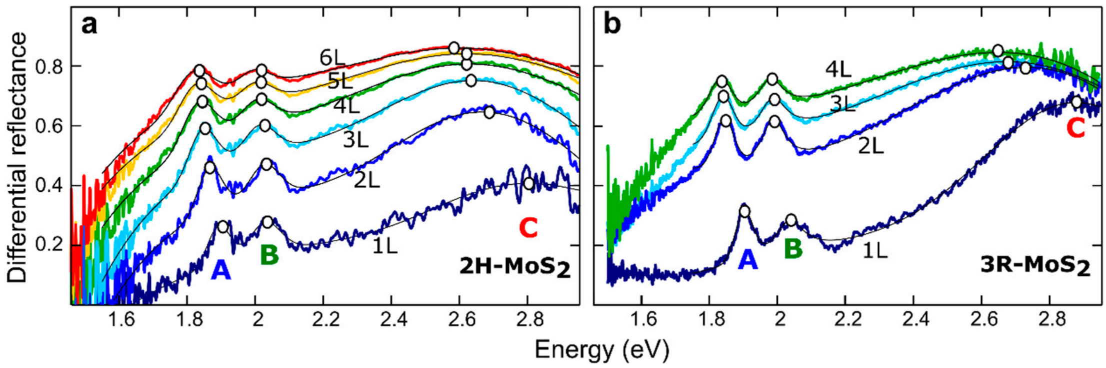

Figure S15. Differential reflectance spectra measured as a function of the number of layers for (a) 2H-MoS_2 (data reproduced from Figure 1d to facilitate the comparison with the 3R polytype), (b) 3R-MoS_2. The spectra have been fitted to a sum of Lorentzian/Gaussian peaks (solid thin black lines) to determine the position of the different excitonic features (highlighted with white circles).

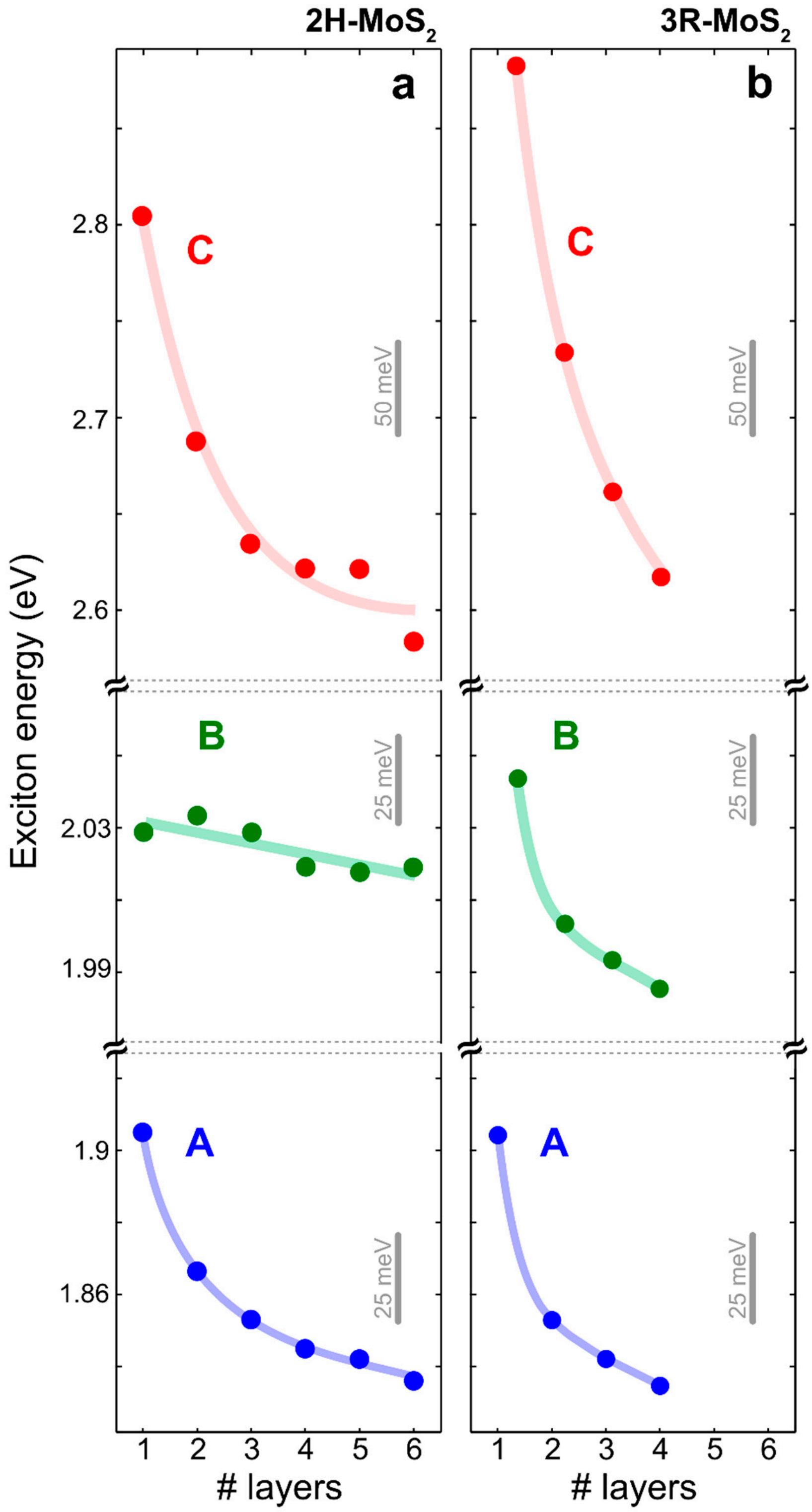

Figure S16. Thickness dependence of the exciton energies, extracted from the differential reflectance spectra of (a) 2H-MoS_2 (data reproduced from Figure 1d to facilitate the comparison with the 3R polytype) and (b) 3R-MoS_2. The solid lines are guides to the eye.

Details on the *ab initio* calculations:

All calculations are performed using a code written on our own [3].

To end up with the absorption spectra of the four different TMDCs, we start with a DFT calculation in the LDA approximation using three shells of localized Gaussian orbitals as basis set. Each of the shells is composed of ten orbital functions covering the symmetries s, p, d and s*. All orbitals inside one shell share the same material dependent decay constant, which are in a range of $0.13\ \mathrm{a_B^{-2}}$ to $2.5\ \mathrm{a_B^{-2}}$. The reciprocal space is sampled with a $12 \times 12 \times 1$ k-point grid for the mono- and bilayers and a $10 \times 10 \times 3$ k-point grid for the bulk crystals. We use the structural parameters as reported in Ref. [4] (for MoS_2 and $MoSe_2$) and Ref. [5] (for WS_2 and WSe_2) with experimental lattice constants of 3.160 Å, 3.299 Å, 3.155 Å and 3.286 Å for MoS_2, $MoSe_2$, WS_2 and WSe_2, respectively. The S or Se atoms of the mono- and bilayer system from neighboring unit cells are vertically separated by at least 28 Å vacuum to suppress interactions due to the periodic continuation perpendicular to the layers (in the DFT). Spin-orbit interaction is included in terms of corresponding pseudopotentials and all spin-split bands enter in the consecutive quasiparticle calculation.

The quasiparticle corrections are calculated within the LDA+GdW [6] approximation, which allows for well converged results at comparably low numerical costs. Figure S17 shows the convergence behavior of the direct gaps at the high symmetry point K with respect to the auxiliary plane wave basis to represent ε and W. For this convergence study, the k-point grid is chosen as $12 \times 12 \times 1$ for the mono- and bilayers and $12 \times 12 \times 3$ for the bulk crystals. The data in Figure S1 show that a plane wave basis of 2.5 Ry (205 plane waves) is already sufficient. At both levels, DFT and GW, the spin-orbit interaction is fully taken into account.

In order to get the absorption spectra we solve the Bethe-Salpeter equation (BSE) using identical k-point grids for the quasiparticle corrections and the electron-hole interactions therefore avoiding the need of an interpolation scheme. The A exciton is the lowest optically bright excitation. The B exciton corresponds to the next optically bright excitation that is not an excited state of the A exciton. Figure S18 and Figure S19 summarize the convergence of the A and B

excitons. Apparently, a k-point grid of 24× 24 × 1 (mono-/bilayer) or 18 × 18 × 3 (bulk) yields well-converged results. These k-point grids are employed for the data shown in Figure S20 and Figure S21. Since the C (D) exciton is composed of several excitations, we calculate the excitation energy as a weighted sum over all excitations inside an energy window that is chosen such that the leading and tailing edges of the peaks are dropped equally to the level of the absorption background (see Figure S20 for more details). To account for uncertainties in the definition of the C (D) exciton, we introduce an error in the respective energetic positions. For the monolayers we include four valence and six conduction bands, while for the bilayer and bulk crystals these numbers are doubled (since the number of atoms in the unit cell are doubled).

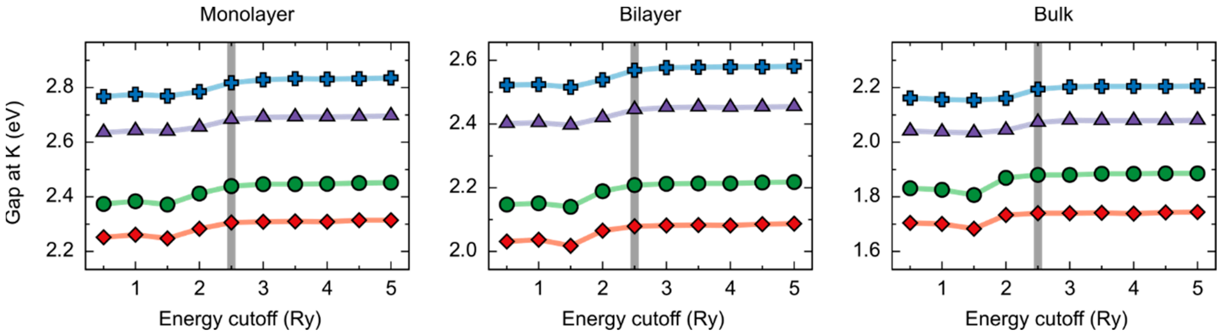

Figure S17. Convergence of the quasiparticle gap at the K point with respect to the energy cutoff used in the LDA+GdW approach for the representation of ε and W. For the mono- and bilayers a k-point grid of 12 × 12 × 1 and for the bulk crystals a k-point grid of 12 × 12 × 3 is used. All four materials MoS_2 (✚), $MoSe_2$ (●), WSe_2 (◆) and WS_2 (▲) show similar convergence behaviour for all three numbers of layers. The grey line shows the chosen energy cutoff of 2.5 Ry employed for preparing the subsequent BSE calculations.

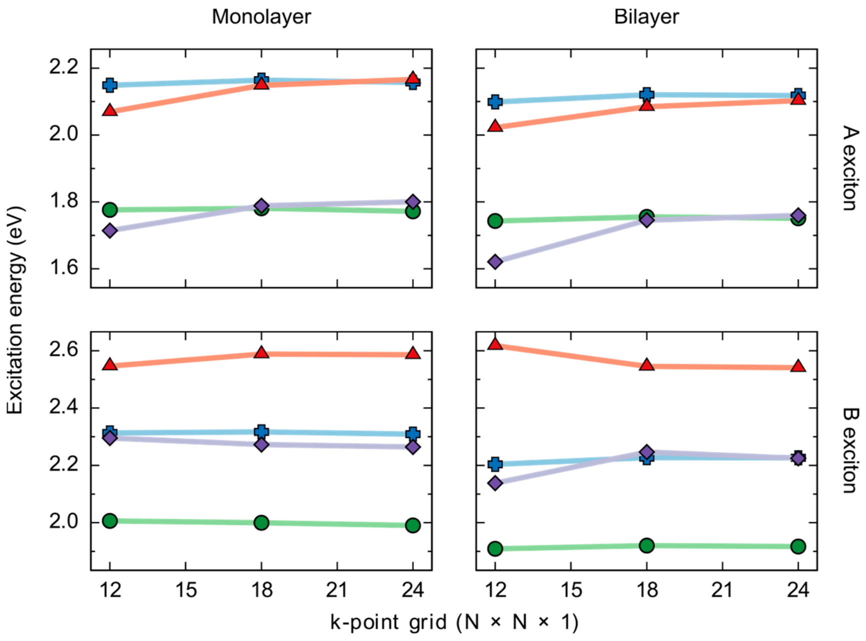

Figure S18. Convergence of the A and B exciton for all four TMDCs MoS_2 (✚), $MoSe_2$ (●), WS_2 (▲) and WSe_2 (◆) with respect to the k-point grid used in the BSE. For the monolayers four valence and six conduction bands are included and for the bilayers eight valence and twelve conduction bands were taken into account. The solid lines are guides to the eye.

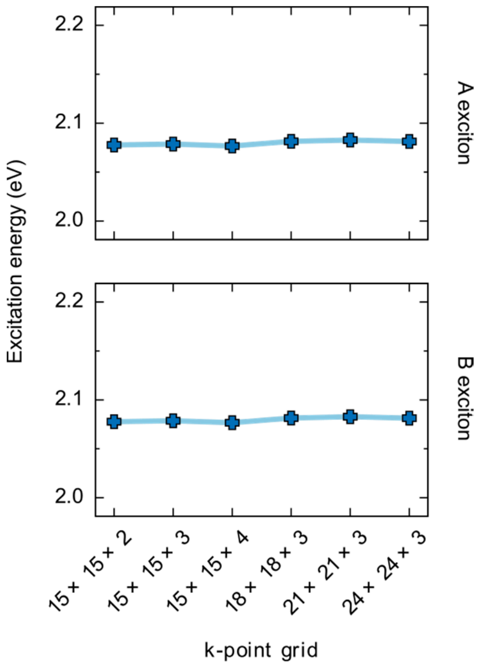

Figure S19. Convergence of the A and B exciton for the bulk crystal of MoS_2 with respect to the k-point grid applied in the BSE. Note that the number of bands were reduced to four valence and six conduction bands for these calculations to facilitate the calculation with $24 \times 24 \times 3$ k-points. The solid lines are guides to the eye.
